# Approximation of bone mineral density and subcutaneous adiposity using T1-weighted images of the human head

**DOI:** 10.1162/imag_a_00390

**Published:** 2024-12-06

**Authors:** Polona Kalc, Felix Hoffstaedter, Eileen Luders, Christian Gaser, Robert Dahnke

**Affiliations:** Structural Brain Mapping Group, Department of Neurology, Jena University Hospital, Friedrich Schiller University Jena, Jena, Germany; Forschungszentrum Jülich, Institute of Neuroscience and Medicine, Brain and Behaviour (INM-7), Jülich, Germany; Institute of Systems Neuroscience, Medical Faculty, Heinrich Heine University Düsseldorf, Düsseldorf, Germany; School of Psychology, University of Auckland, Auckland, New Zealand; Department of Women’s and Children’s Health, Uppsala University, Uppsala, Sweden; Laboratory of Neuro Imaging, Keck School of Medicine, University of Southern California, Los Angeles, CA, United States; Department of Psychiatry and Psychotherapy, Jena University Hospital, Friedrich Schiller University Jena, Jena, Germany; German Center for Mental Health (DZPG), Berlin, Germany

**Keywords:** structural MRI, skull-stripping, cranium, bone mineral density, adiposity, UK Biobank

## Abstract

Bones and brain are intricately connected and scientific interest in their interaction is growing. This has become particularly evident in the framework of clinical applications for various medical conditions, such as obesity and osteoporosis. The adverse effects of obesity on brain health have long been recognised, but few brain imaging studies provide sophisticated body composition measures. Here, we propose to extract the following bone- and adiposity-related measures from T1-weighted MR images of the head: an approximation of skull bone mineral density (BMD), skull bone thickness, and two approximations of subcutaneous fat (i.e., the intensity and thickness of soft non-brain head tissue). The reliability and validity of these four distinct measures were tested in two large-scale databases, the UK Biobank and OASIS-3. The measures pertaining to skull BMD, skull bone thickness, and intensity-based adiposity proxy proved to be reliable (ICC = .95/.83/.66,*p *< .001) and valid, with high correlations to DXA-derived head BMD values (rho = .70,*p*< .001) and MRI-derived abdominal subcutaneous adipose volume (rho = .62,*p*< .001). Thickness-based adiposity proxy had only a low retest reliability (ICC = .53,*p*< .001). The outcomes of this study constitute an important step towards extracting relevant non-brain features from available brain scans.

## 1 Introduction

The brain is closely connected to other tissues and organs (W. Zhou et al., 2023), including the skeletal system. A large body of literature suggests a bidirectional communication between bones and the brain (Obri et al., 2018;Rousseaud et al., 2016). Moreover, bone-derived metabolites, such as osteocalcin, have been implicated in mood, cognition, and glucose/energy homeostasis in murine models (Guntur & Rosen, 2012;Khrimian et al., 2017;N. K. Lee et al., 2007;Nakamura et al., 2021). However, the neuroimaging community largely dismisses the information from the closest bone structure surrounding the brain.

Bone tissue undergoes constant remodelling which is supported by osteoclasts (i.e., bone resorbing cells) and osteoblasts (i.e., new bone-forming cells) to regulate mineral homeostasis and ensure skeletal integrity (Clarke, 2008;Feng & McDonald, 2011). Bone metabolism is affected by endocrine signalling and innervation via the sympathetic nervous system, with greater sympathetic activity leading to decreasing BMD (Ducy & Kousteni, 2015;Takeda et al., 2002). Furthermore, bone tissue actively signals to the brain and other organs and is implicated in brain metabolism by secretion of bone-derived peptides (e.g., osteocalcin, osteopontin, sclerostin, lipocalin-2, etc.), as well as by production of bone marrow-derived immune cells (Chen et al., 2021;Yu et al., 2020).

**Fig. 1.**
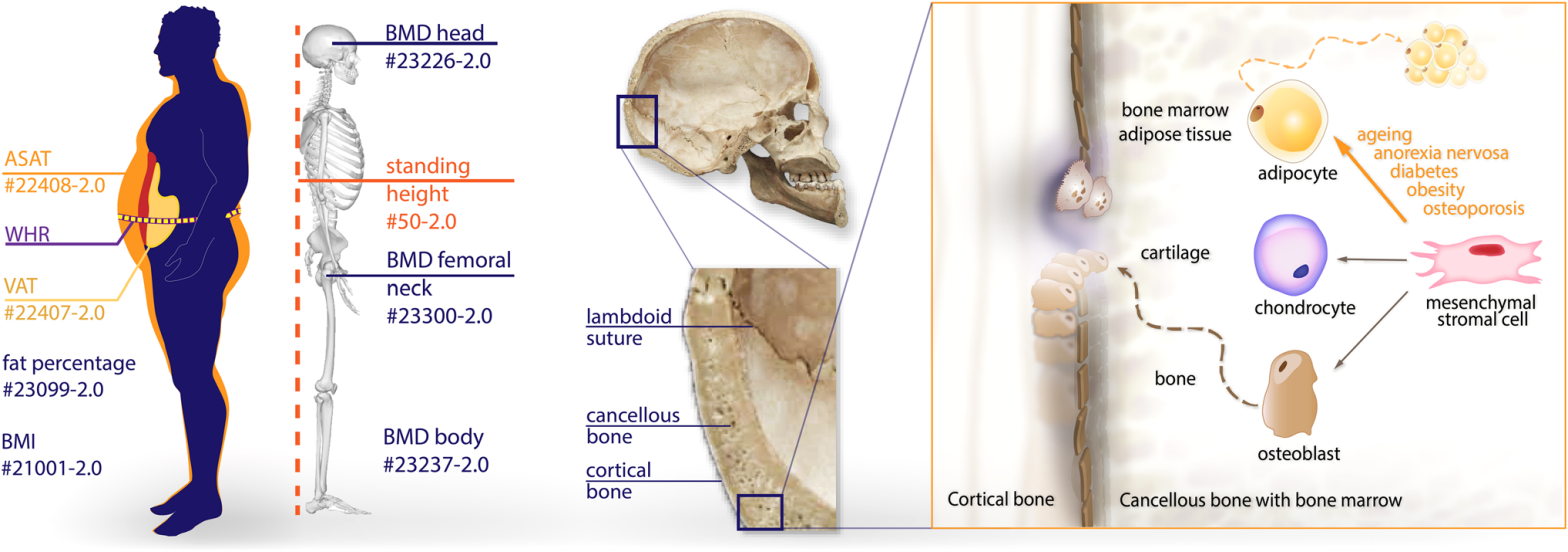
On the left are the UK Biobank (UKB) anthropometric measures used in the validation process (ASAT: abdominal subcutaneous adipose tissue, WHR: waist to hip ratio, VAT: visceral adipose tissue, BMI: body mass index, BMD: bone mineral density). Cranial anatomy and microenvironment of a bone are presented on the right. Bone marrow is located within the spongy/cancellous bone (diploë). Mesenchymal stromal cells in the bone marrow have the potential to develop into osteoblasts (bone-forming cells), chondrocytes (cartilage-forming cells), or adipocytes (bone marrow fat cells). Obesity, ageing, diabetes, anorexia nervosa, starvation, and osteoporosis lead their differentiation into adipocytes and, hence, a lower BMD (Tencerova & Kassem, 2016).

Bone health is compromised in various metabolic diseases, and the relation between BMD and fat mass is complex (N. J. Lee et al., 2020;Rinonapoli et al., 2021). For example, people with anorexia nervosa tend to have lower BMD than healthy individuals or people with obesity (Fazeli & Klibanski, 2014;Maïmoun et al., 2020). However, increased adiposity levels or other metabolic disorders can have a detrimental effect on bone health, as the mesenchymal stromal cells of the bone marrow preferentially convert into adipocytes (i.e., fat cells) rather than osteoblasts (i.e., new bone-forming cells) in obesity, potentially resulting in reduced bone mass (Fig. 1) (Ambrosi et al., 2017;Veldhuis-Vlug & Rosen, 2018). Bone health also seems to play a role in neurodegenerative as well as neurodevelopmental disorders (Kelly et al., 2020), such as Parkinson’s disease (Sleeman et al., 2016;Tassorelli et al., 2017), multiple sclerosis (Bisson et al., 2019), and autism spectrum disorder (Neumeyer et al., 2015). Moreover, a low BMD has been associated with an increased risk of Alzheimer’s Disease (Kostev et al., 2018;Lui et al., 2003;Tan et al., 2005;Xiao et al., 2023;Zhang et al., 2022;R. Zhou et al., 2011). Therefore, bone-related measures may be of significant interest to the neuroimaging community.

Given the consistent reports on the connection of bone measures (e.g., BMD, bone mineral content) and brain ageing in the UK Biobank database (e.g.,Miller et al., 2016;Smith et al., 2019,^2020^) as well as the long-known adverse effects of obesity on cognitive and brain health (Beydoun et al., 2008;Farruggia & Small, 2019), the availability of such measures in open-source brain imaging databases, such as IXI, ADNI, AIBL, or OASIS, is inadequate. Many of the aforementioned databases include body composition proxies, such as BMI, but lack sophisticated body composition and bone-related measures, which typically require other means of acquisition (e.g., DXA-scanning, heel bone ultrasound, high-resolution peripheral quantitative computed tomography). There has been a surge of interest in the crosstalk between bones and brain as well as adiposity in recent years, and prospective studies are likely to incorporate the acquisition of relevant data in the future. However, in the meantime, studies may benefit from (retrospective) approximations of such measures using already existing neuroimaging data.

Thus, here we present an approach to extract relevant measures—bone mineral density (BMD), skull bone thickness, and two approximations of subcutaneous fat, specifically the intensity and thickness of soft non-brain head tissue—from non-brain tissue classes that are normally discarded when processing T1-weighted images of the head. Standard processing tools for structural MR images (e.g., SPM) contain algorithms that allow for identifying bone and soft tissue segments. The proposed approach builds on these established and well-tested algorithms but extends them by incorporating the extraction (and evaluation) of relevant skull and soft tissue measures.

## 2 Methods

Section 1 describes the development of the algorithm to extract the novel measures from T1-weighted MRI images of the head, whereasSection 2describes the evaluation of the reliability and validity of the novel measures. BothSection 1and2used data from the UK Biobank (UKB application #41655) and from the OASIS-3 Longitudinal Multimodal Neuroimaging Study (OASIS-3), but to different extents and for different purposes (as detailed below).

The UKB has ethical approval from the North West Multi-Centre Research Ethics Committee (MREC) and is in possession of the informed consents. The OASIS-3 has ethical approval from the Washington University Human Research Protection Office and is in possession of the informed consents. The public data sharing terms for OASIS-3 were also approved by the Washington University Human Research Protection Office.

## Section 1

### 2.1 Data sources

To develop the four novel head measures, we used T1-weighted brain MR images of a subsample of 1000 healthy UKB subjects (M_Age_= 64.02 ± 6.40 years, age range: 46–80 years, 50% women). In addition, we used 114 CT scans from the OASIS-3 dataset (M_Age_= 66.71 ± 8.38 years, age range: 42–85, 51% women) to create a skull-segment atlas.

### 2.2 Data acquisition

T1-weighted scans for the UKB were acquired on Siemens Skyra 3.0 T scanners, as detailed elsewhere (https://biobank.ctsu.ox.ac.uk/crystal/crystal/docs/brain_mri.pdf). In short, images were obtained with a 32-channel RF receive head coil using an MPRAGE sequence (1-mm isotropic resolution, inversion/repetition time = 880/2000 ms, acquisition time: 5 minutes, FOV: 208 x 256 x 256 matrix, in-plane acceleration factor = 2). The quality of the scans was monitored internally through the UKB workflow (Alfaro-Almagro et al., 2018).

CT scans for the OASIS-3 dataset were obtained on the Siemens Biograph 40 PET/CT scanner. A 3-second X-ray topogram was acquired in the lateral plane, and a spiral CT scan was performed for attenuation correction at the low-dose CT (LaMontagne et al., 2019).

### 2.3 Data preprocessing

The T1-weighted images were processed using SPM12 (Wellcome Center for Human Neuroimaging,https://www.fil.ion.ucl.ac.uk/spm/) running under Matlab 2021a (Mathworks Inc., Natick, MA, USA), which produced the following segments: grey matter, white matter, cerebrospinal fluid, skull, soft head tissue, and background based on tissue probability map (TPM;Ashburner & Friston, 2005). Of note, instead of the default setting (3 mm), we set SPM’s “samp” parameter to 5 mm to ensure that non-brain tissues were properly classified.

The CT images were preprocessed with CTseg utilising the unified segmentation of SPM12 (Brudfors et al., 2020;https://github.com/WCHN/CTseg) and co-registered to the individual MRI space.

### 2.4 Extraction of the novel head measures

We extracted head features from T1-weighted MRI images in two ways: (1) separated by image class (hard vs. soft non-brain head tissues) and (2) by measure type (intensity vs. thickness). The hard head tissue (i.e., bone) is used to quantify BMD, whereas the soft head tissue serves to approximate the adiposity. The intensity-based measures are more sensitive but depend on the MR protocol. Thickness-based measures are less protocol-dependent as they rely more on the segmentation, but do not allow further specification of tissue composition (e.g., quantification of adiposity within the tissue compartment). In both cases, estimating global/regional aspects is important to reduce local dependencies and errors.

#### 2.4.1 Correction of SPM segmentation

**Fig. 2.**
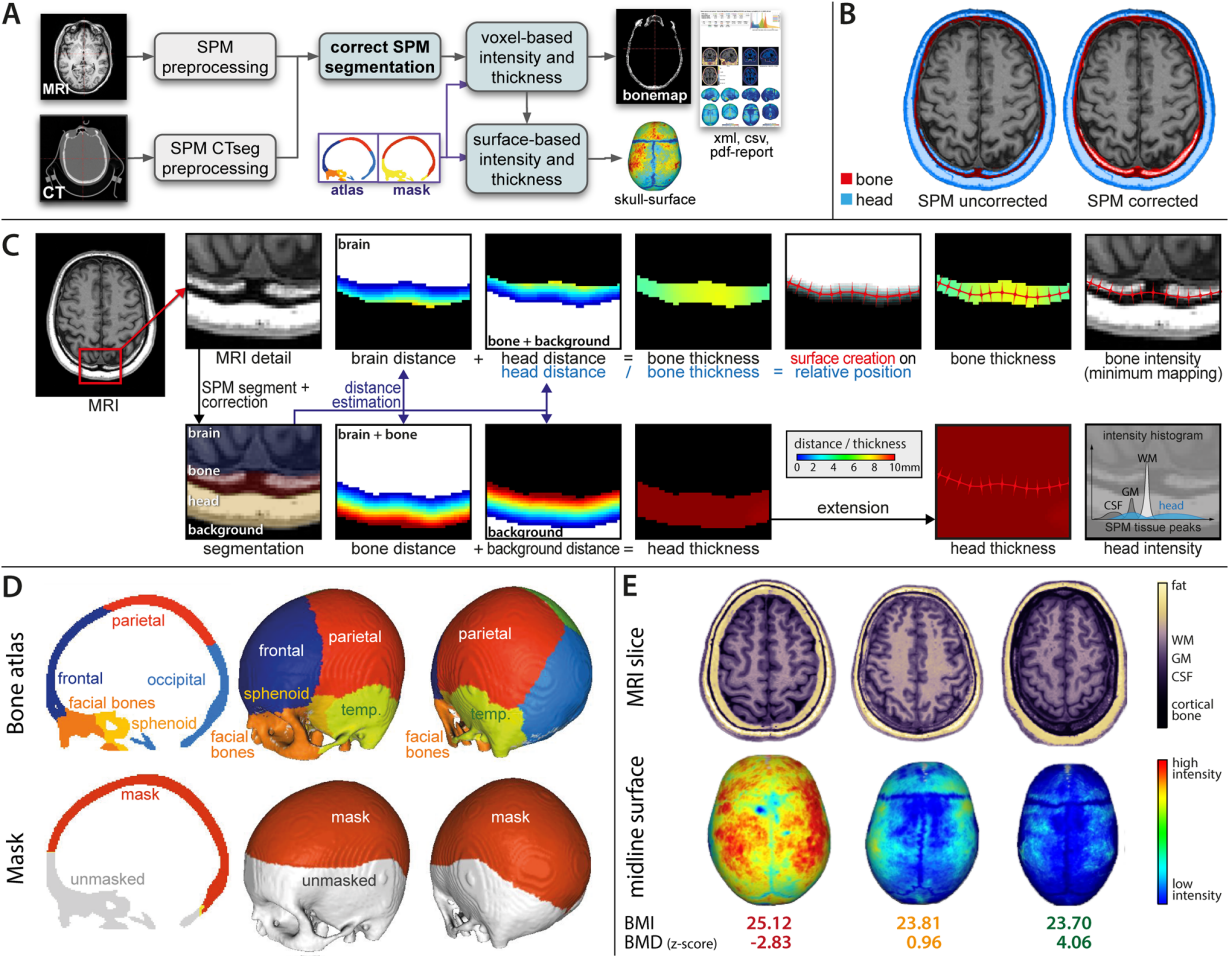
(A) The figure shows the workflow of the algorithm with necessary corrections (B), extraction of measures (C), and template maps (D). MRI/CT images undergo preprocessing by SPM/SPM-CTseg into CSF, GM, WM, skull, head, and background. Morphological operations are used to correct segmentation errors (B), before estimating various distance maps to describe the skull and head tissue thickness maps (C). The calvarial atlas and mask, shown in (D), are mapped to the individual space to extract regional and global values. If surface processing is required, the distance maps are used to create a percentage position map, which allows the creation of the skull’s midline surface. This surface is then used to map the local thickness, intensity, and atlas region to the surface to extract a regional or masked global thickness and intensity value. (E) Represented are MRI slices and skull surfaces of three 63-year-old women from UKB with similar BMI but varying degrees of head BMD. Low BMD is associated with increased intensity of the skull segments in T1-weighted images.

The SPM processing often erroneously assigned the high intensity bone marrow in the cancellous bone to the head tissue class instead of the skull segment. Our algorithm automatically corrected misclassified skull segments by morphological operations (e.g., erosion, dilation, closing, opening, labelling) to avoid missing the intensities from the diploë (i.e., cancellous/spongy bone between the inner and outer layer of the cortical bone of the skull) and to correct the underestimated bone thickness (Fig. 2B).

#### 2.4.2 Boney atlas

We created a template atlas of the skull regions by averaging affine and intensity-normalised T1-weighted images as well as CT data of OASIS-3, and manually labelling the skull segment tissue probability map using Slicer3D (Fedorov et al., 2012;https://slicer.readthedocs.io/en/5.0/index.html). The atlas was mapped into individual space using the linear transformation from the SPM segmentation. Additionally, we created a bone mask to avoid critical regions that are often affected by defacing or are in parts very thin, such as temporal and sphenoid bones (Fig. 2D). The identified bone regions were used in extracting regional bone and adiposity measures.

#### 2.4.3 Bone measures

##### 2.4.3.1 Intensity-based measures

Bone measures were derived from the corrected SPM skull segment, by quantifying the mean intensity across the entire segment labelled as skull (or within a specific skull region), and normalised by the SPM-derived WM intensity.

##### 2.4.3.2 Thickness-based measures

Bone thickness was defined as the sum of the shortest distance from each skull voxel to the CSF and to the soft non-brain head tissue.

In addition to voxel-based processing, a midline surface was generated to map bone values along the surface-normal. This allowed the cortical bone intensity, quantified as the local minimum, to be separated from the bone marrow intensity, defined as the distance-weighted average of the mapped points (Fig. 2C).

#### 2.4.4 Soft head tissue measures

##### 2.4.4.1 Intensity-based measures

To approximate the adipose tissue of the head, we estimated a weighted average of the SPM-derived Gaussian peaks by their proportions in the tissue class labelled as head. Head tissue intensity was described as a percentage composite of the various soft non-brain head tissues.

To be more precise, SPM uses various Gaussian priors to model the composition of each tissue class. The Unified Segmentation (Ashburner & Friston, 2005) utilises a tissue probability map with 6 classes (GM, WM, CSF, hard head tissue, soft head tissue, and background) and various number of Gaussian priors that represent the most relevant intensity peaks within the region outlined by the TPM (Fig. 2Chistogram). Gaussian peaks are therefore highly dependent on the tissue composition, but do not necessarily represent particular tissues. Hence, our intensity-based measure is based on the average intensity and the size of the non-brain “soft head tissue” Gaussians, described by standard deviation and a percentage volume that we used for weighting. Subjects with greater estimated adipose levels have therefore a higher relative volume of high-intensity voxels (in protocols without fat suppression).

##### 2.4.4.2 Thickness-based measures

Local head thickness was calculated as the shortest distance from each soft head tissue voxel to the skull and to the background. The sum of both distances then yielded the estimate of the voxel-wise head tissue thickness. A separation into different head tissues (i.e., muscle, skin, and fat) was omitted because of varying amounts of (chemical shift) artefacts and inhomogeneities across the image. Of note, the lower portions of all brain scans as well as voxels located more than 30 mm from the skull were excluded from the head tissue thickness estimation to avoid side effects due to defacing and/or varying scanning protocols and procedures.

### 2.5 Selection of the best proxy measures

Extracted global and regional measures were tested on 1000 UKB subjects to obtain the best estimates of the BMD in an exploratory manner. A random forest regression with all the newly extracted bone measures as independent variables predicting the gold-standard UKB head’s BMD measure and a permutation test were run in R (version 4.3.3;R Core Team, 2024) with packages*party*(Hothorn et al., 2006) and*flexplot*(D. Fife, 2022). The final BMD approximation measure was chosen based on the estimated predictor importance values from a permutation test (D. A. Fife & D’Onofrio, 2023).

The results of this step showed that the most relevant measure for the BMD approximation is the mean intensity extracted from the occipital bone (Table S1). The results were confirmed by the VBM analysis of the skull segments, where we could see the highest association of the head BMD and the occipital region of the skull (Fig. 4). We assumed the same pattern for the head tissue thickness and intensity estimates based on visual inspection of the images. All further analyses therefore included the occipital measures to represent the skull BMD, skull bone thickness, intensity-based (IAP), and thickness-based adiposity proxies (TAP).

## Section 2

### 2.6 Data sources

T1-weighted brain MR images of a subsample of 1000 healthy UKB subjects that were not included in the previous exploratory analysis (M_Age_= 63.81 ± 6.27 years, age range: 46–79 years, 50% women) were used to validate our skull BMD and adiposity proxy measures.

Furthermore, we used a sample of 316 T1-weighted MR scans from OASIS-3 dataset, acquired at two time points within a time interval of less than 3 months (M_Age_= 67.66 ± 8.22 years, age range: 42–85, 56% women) to determine the retest reliability of the measures for the same as well as (slightly) different scanners and protocols.

### 2.7 Data acquisition

#### 2.7.1 Brain MRI data acquisition

Please refer to theSection 1above regarding the acquisition of T1-weighted brain MR images in UKB.

The MRI data from the OASIS-3 dataset used in this study were acquired on different 1.5 T and 3.0 T scanners with a 20-channel head coil (Siemens Sonata, Siemens TIM Trio and BioGraph mMR PET-MR 3T) (LaMontagne et al., 2019). The MPRAGE sequence was used with (i) 1 mm isotropic repetition/echo time = 1900/3.93 ms, FA = 15° FOV phase = 87.50%; (ii) 1 mm isotropic inversion/repetition/echo time = 1000/2400/3.16 ms, FA = 8°, FOV phase = 100%, and R = 2; and (iii) 1.20 x 1.05 x 1.05 mm resolution, inversion/repetition/echo time = 900/2300/2.95 ms, FA = 9°, FOV phase = 93.75 mm, and R = 2.

#### 2.7.2 Bone mineral density data acquisition

BMD measures of the head, left femoral neck, and total body were obtained by dual energy X-ray absorptiometry (DXA) scanning with iDXA instrument (GE-Lunar, Madison, WI). A detailed overview of the acquisition procedure is available in UK Biobank documentation (https://biobank.ndph.ox.ac.uk/showcase/ukb/docs/DXA_explan_doc.pdf).

#### 2.7.3 Abdominal adipose tissue measures acquisition

UK Biobank’s subcutaneous and visceral adipose tissue volumes of the abdomen were acquired by abdominal MRI imaging on Siemens MAGNETOM Aera 1.5 T MRI scanner (Siemens Healthineers, Erlangen, Germany) with a 6‐minute dual‐echo Dixon Vibe protocol, resulting in a water and fat separated volumetric data set (Linge et al., 2018) that was processed by AMRA Profiler Research (AMRA Medical AB, Linköping, Sweden) to obtain body composition measures (Borga et al., 2015).

### 2.8 Head MRI data preprocessing

The brain MRI data in this section were processed with the newly developed algorithm as described above (Fig. 2A). Briefly, SPM12-derived skull and soft head tissue segments were corrected by morphological operations. The calvarial atlas and mask were mapped to the individual space to extract regional and global values for the voxel-based measure extraction. In the surface-based extraction, distance maps were used to create a percentage position map, forming the mid-skull-surface. This was used to map the local thickness, intensity, and atlas region to the surface and extract a regional or masked global thickness and intensity value.

### 2.9 Validation of the novel measures

#### 2.9.1 Examination of validity (UK Biobank dataset)

The segmentation results were visually examined against ground-truth CT data, juxtaposing MR- and CT-derived measures from the OASIS-3 dataset (seeFig. S1).

The selected measures were validated by calculating the Spearman correlation coefficient (*psych*package;Revelle, 2024) with head BMD (UKB #23226-2.0) and total body BMD (UKB #23239-2.0), as well as body fat percentage (UKB #23099-2.0) and abdominal subcutaneous adipose tissue (ASAT; UKB #22408-2.0) volume, for the estimates of skull BMD and head tissue thickness, respectively (seeFig. 1).

We additionally inspected the relation to other anthropometric and lifestyle variables available in the UKB dataset that had previously been connected to BMD or adiposity (Cusano, 2015;Molenaar et al., 2009;Shojaa et al., 2020;Ward & Klesges, 2001;Williams, 2005). We investigated the association with the BMD of the left femoral neck (UKB #23300-2.0), standing height (UKB #50-2.0), BMI (UKB #21001-2.0), and visceral adipose tissue volume (VAT; UKB #22407-2.0). We also used waist circumference (UKB #48-2.0), hip circumference (UKB #49-2.0), and height (UKB #50-2.0) to derive and test the association with the waist-to-hip ratio (WHR) and waist-to-height ratio (WHtR), which may better reflect obesity-related cardiovascular risk independent of age, sex, or ethnicity (Ashwell, 2005;Swainson et al., 2017). Furthermore, we included the variables connected to physical activity, namely duration of moderate (UKB #894-2.0) and vigorous physical activity (UKB #914-2.0), as well as pack years of smoking as proportion of life span exposed to smoking (UKB #20162-2.0) and frequency of alcohol intake (UKB #1558-2.0).

As an additional validation step, we performed a volume-based morphometry (VBM) analysis in SPM12 with BMD of the head and our newly extracted proxy BMD as predictors of the extracted warped and smoothed (FWHM = 8 mm) intensity-normalised skull segments on the sample of 2000 UKB subjects (M_Age_= 64.17 ± 6.34 years, age range: 46–89 years, 50% women). Furthermore, a logistic regression analysis was conducted to examine the relationship between the occurrence of fractures resulting from a simple fall (UKB #3005-2.0,*n*fractures = 116) and our proxy BMD measure. The model was fitted using the*glm*function in R (version 4.3.3;R Core Team, 2024) with age and sex as covariates.

#### 2.9.2 Evaluation of reliability (OASIS-3 dataset)

We calculated the retest reliability of the estimated measures using T1-weighted images from the OASIS-3 dataset acquired at two time points within an interval of less than 3 months. One subject was excluded from the analysis due to a failed SPM-segmentation, resulting in a total sample of 157 subjects (age range: 42–85, M_Age_= 67.64 ± 8.24 years, 55% women). The reliability of the measures was estimated under the same scanner/protocol and mixed scanner/protocol conditions using an intraclass-correlation coefficient (2-way-mixed-effect model, absolute agreement) from the*psych*package (Revelle, 2024).

Furthermore, due to different resolution in some of the protocols, we harmonised the data to remove the batch effects employing ComBat (Johnson et al., 2007) in*sva*package (Leek et al., 2023).

## 3 Results

### 3.1 Validation of the measures on the UK Biobank dataset

**Fig. 3.**
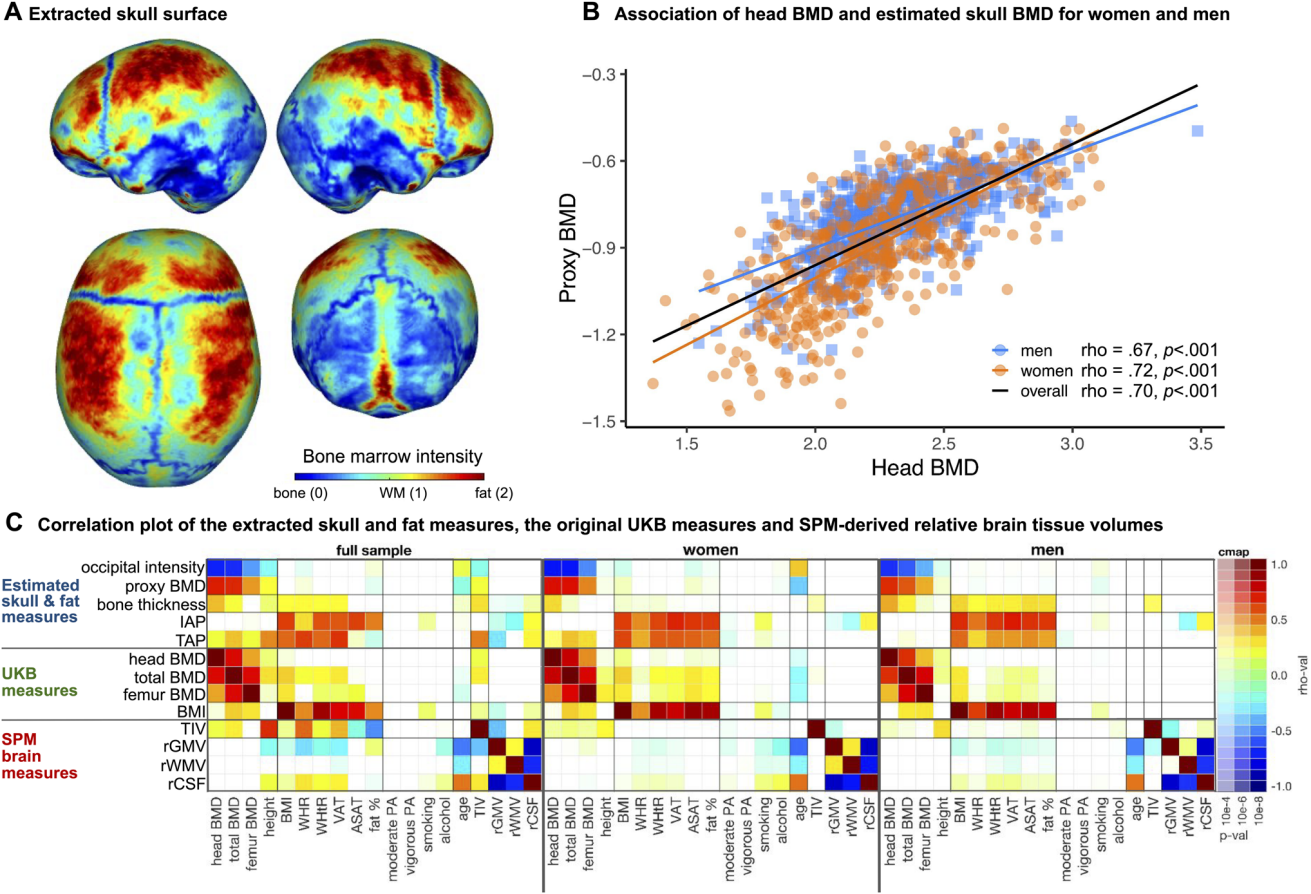
(A) The extracted skull surface of a subject from the UK Biobank with visible sutures and high intensity bone marrow. (B) Scatterplot of the UKB head BMD and our skull BMD estimate for men and women. Higher head BMD is related to lower (occipital) bone intensity in the MRI and therefore results in a higher proxy BMD value. (C) Spearman correlation coefficients with Holm correction for multiple comparisons between the estimated skull BMD proxy, bone thickness, as well as intensity- and thickness-based adiposity proxies (i.e., SPM head class and head thickness, respectively), and other UKB and brain measures. The extracted measures are associated with the original BMD as well as body composition measures.

The processing results yielded clearly identifiable coronal, sagittal, and lambdoid sutures, visibly separating the calvarial bones in our extracted skull surfaces (Fig. 3A). The amount of high intensity voxels in the skull is negatively associated with BMD (proxy) (Fig. 2E).

**Fig. 4.**
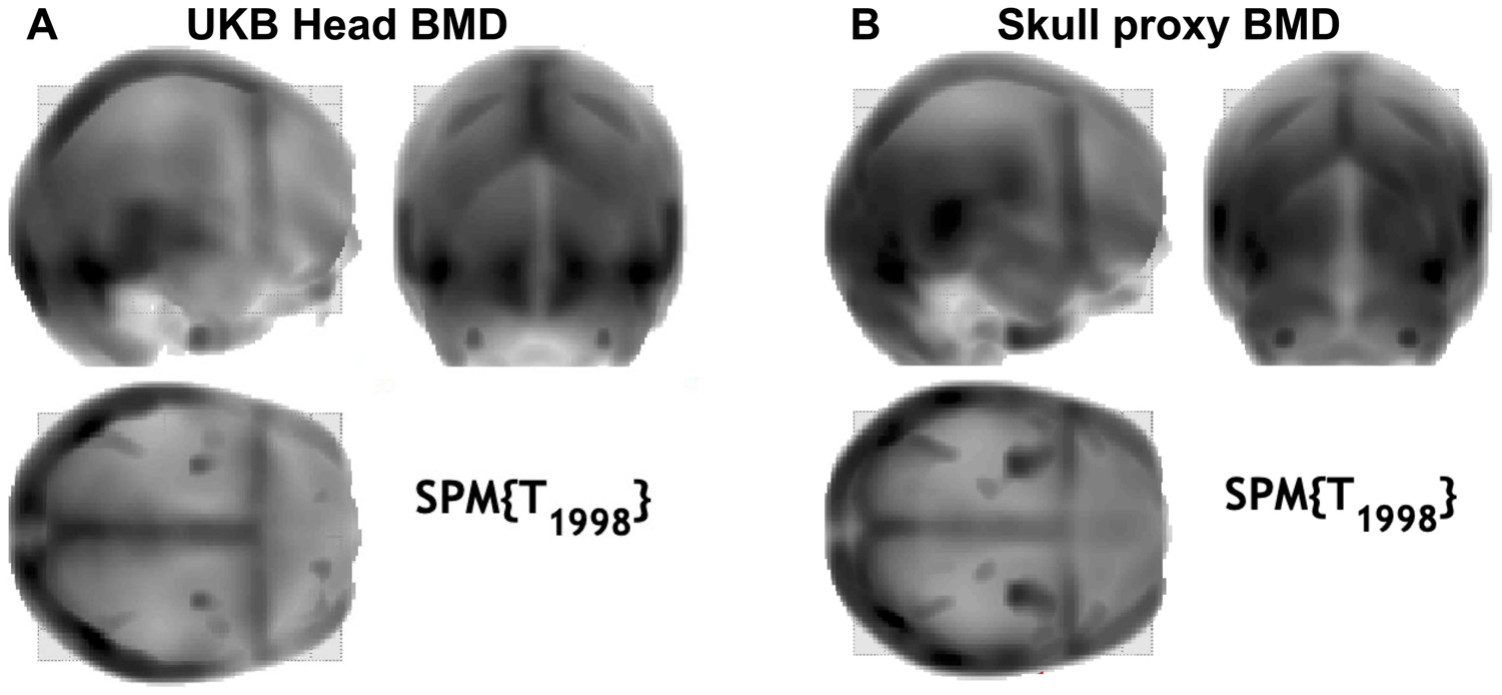
“Glass” head maps from the VBM analysis in SPM with (A) head BMD from the UKB as a predictor of the intensity-normalised skull segments, and (B) proxy BMD measure as a predictor of the intensity-normalised skull segments**.**

The validation results against other anthropometric and lifestyle measures are shown inFigure 3C(andFigs. S2–S6). The extracted measures showed valid associations to other UKB parameters. The Spearman’s correlation coefficients between the extracted proxy BMD measure and UKB-derived head BMD were .70, .72, and .67 for a total sample, women, and men, respectively (Fig. 3B;Fig. S3for the bootstrapped correlation coefficient). The correlation with the total body BMD was moderate for the total sample and the subsample of men (rho = .64 and .57, respectively) and higher for women (rho = .72). The association to other anthropometric measures showed a weak negative correlation with total fat percent (rho = –.14,*p*< .01), and a low positive correlation with WHR (rho = .14,*p*< .01). A weak negative association between the proxy BMD measure and the exposure to smoking was observed in a subsample of men (rho = –.12,*p*< .01). Bone thickness (in the occipital part) was, on the other hand, weakly associated with body composition measures in men (ASAT: rho = .31,*p*< .0001; WHR: rho = .24,*p*< .0001, percent body fat: .30,*p*< .0001).

Our intensity-based adiposity approximation was moderately to highly positively correlated with other body composition measures. The Spearman’s correlation coefficients with BMI were .52, .52, and .57 for a total sample, women, and men, respectively. The association is higher for the waist to height ratio (WHtR; rho = .59; rho = .62; rho = .64), volume of visceral adipose tissue (VAT), namely .57, .68, and .70, and abdominal subcutaneous adipose tissue volume (ASAT; rho = .62; rho = .64; rho = .65), for a total sample, women, and men, respectively. Similar patterns were observed in our thickness-based adiposity approximation. The measure was moderately positively associated with other body composition measures. The Spearman’s correlation coefficients with BMI and WHtR were moderate (BMI: .50, .54, .48, WHtR: .55, .57, and .44), whereas the association with VAT volume was higher, namely .65, .56, and .45 for a total sample, women, and men, respectively. The thickness-based adiposity proxy had a similar correlation with VAT as BMI in a total sample (rho = .65,*p*< .0001; rho = .64,*p*< .0001 for VAT association with TAP and BMI, respectively), but lower in subsamples of men and women (seeFig. S2).

It could be observed that the measures were biased due to anthropometric differences between the biological sexes. For subsequent use in statistical analyses, the sex, age, and TIV of the participants must be taken into account.

The logistic regression model showed a significant association between proxy BMD and fracture occurrence (OR = .19, 95% CI [.08 .47],*p*< .001). Higher levels of proxy BMD and being male (OR = .40, 95% CI [.25 .62],*p*< .001) were associated with lower odds of having experienced a fracture from a simple fall, while age did not seem to significantly affect the odds (Table 1).

**Table 1:** Results of a logistic regression model.

	Estimate	Odds ratio	Lower CI 95%	Upper CI 95%	*p* -value
Intercept	-4.293	0.014	0.002	0.107	<.001 ***
Proxy BMD	-1.659	0.190	0.077	0.472	<.001 ***
Sex (male)	-0.929	0.395	0.253	0.616	<.001 ***
Age	0.005	1.004	0.973	1.037	.783

*** The significance (as stated) is*p* < .001.

### 3.2 The reliability of the measures in OASIS-3 dataset

The retest reliability based on the data obtained with the same protocol on two different time-points (*n*= 63, M_Age_= 69.27 ± 7.32 years, 48–85, 54% women) was high for the skull BMD (*ICC*= .95;*p*< .001) and bone thickness estimation (*ICC*= .83,*p*< .001), comparatively to the regional GM volume (*ICC*= .97,*p*< .001). Our thickness-based adiposity proxy showed lower retest reliability (*ICC*= .53,*p*< .001). Nevertheless, the intensity-based adiposity proxy had a sufficient reliability of .66 (*p*< .001). It should be noted that the retest reliability of the measures derived from images acquired with different protocols is generally lower than for identical protocols. The results from the whole selected sample (*n*= 157) as well as the results on the harmonised data are available inTable S2.

## 4 Discussion

In the present study, we used tissue classes that are typically discarded when processing brain images to estimate skull BMD, skull bone thickness, as well as intensity- and thickness-based adiposity approximations. Such measures are normally not considered in neuroimaging research. However, burgeoning research shows the interconnectedness between brain, bones, and adipose tissue (Caron et al., 2018;Rousseaud et al., 2016). Thus, the aim of this new line of research is to provide the neuroimaging community with proxy measures that can be extracted from standard T1-weighted brain scans that are available in open-source databases or that have been acquired by researchers following standard protocols of brain imaging.

### 4.1 Reliability and validity of measures

All extracted measures showed a good retest reliability on the OASIS-3 dataset in both same- and mixed-protocol test cases after harmonisation, with the only exception of thickness-based adiposity proxy. Its low retest reliability might be due to bias introduced during the head segment correction, and further development is currently underway to improve this specific measurement.

The validity of the proxy BMD measure was confirmed by moderate to high associations with DXA-derived BMD measures of the head and whole body, and by its association with having experienced a fracture due to a simple fall. The proxy BMD measure also showed a link to age in women (but not men). This is expected as women experience significant changes in bone mass with ageing due to decreases in oestrogen and increases of follicle-stimulating hormone (Almeida, 2012;Iqbal et al., 2012). Men, on the other hand, are less susceptible to age-related hormonal fluctuations, and their bone mass tends to remain stable until later in life (Porter & Varacallo, 2022). However, certain medications and poor lifestyle choices (e.g., physical inactivity or smoking) can also increase the risk of developing osteoporosis in men (Porter & Varacallo, 2022). In fact, our proxy BMD measure was partly linked to smoking exposure in men.

The validity of skull bone thickness was partially confirmed by association with certain aspects of body composition measures, particularly in men. Higher BMI and abdominal adiposity were weakly related to higher skull bone thickness in the occipital region. These results could be linked to the body weight-related mechanical stress to the bone (Cherukuri et al., 2021). It has been shown that men have, in general, a thicker occipital cranial vault (Anzelmo et al., 2015), related to abundant muscle attachments in that region (De Boer et al., 2016). Since it is typically the lean body mass that has a positive effect on bone health, rather than fat mass (Ho-Pham et al., 2014;Nguyen et al., 2020), our findings are to some extent contradictory. Nevertheless, the connection between skull bone thickness and adiposity might involve the effects of adipokines (i.e., signalling molecules produced by the adipocytes, such as leptin) on bone mass (Mangion et al., 2023); however, further studies are needed to elucidate this finding.

The associations of thickness- and intensity-based adiposity proxy measures with UKB anthropometric measures were moderate to high, with the lowest correlation to BMI and the highest to WHtR and VAT. This lower connection to BMI is not surprising, as BMI is inherently a proxy measure of body composition (Gutin, 2018). Among other drawbacks, BMI fails to account for changes in body composition when ageing, where lean muscle mass typically decreases while visceral adiposity increases, resulting in no change in weight (Bhurosy & Jeewon, 2013). In an ageing sample like the UKB, such limited association to BMI is expected. Nevertheless, a stronger association between thickness-based adiposity proxy with VAT (in comparison to BMI) is crucial, as it shows the relevance of its extraction for the approximation of abdominal obesity. Moreover, both adiposity proxy measures were also negatively correlated with the relative GM volume and positively with CSF volume, which corroborates the previous findings on the negative influence of obesity on brain structure (Gómez-Apo et al., 2021).

### 4.2 Potential application

Previous studies have shown that head fat tissue volume is linked to estimates of body composition in people with obesity (Wang et al., 2014). Nevertheless, to our knowledge, we conducted the first study that provides an estimate of BMD from a T1-weighted scan of the head. The outcomes of our study suggest that skull BDM can serve as a proxy measure of a person’s total BMD. In addition, the information on skull bone thickness might become relevant in studies of traumatic brain injuries as the cranium plays a vital role in protecting the brain (Semple & Panagiotopoulou, 2023). Furthermore, the thickness-based adiposity proxy measures can be included as potential confounds in functional neuroimaging studies, such as electroencephalography, functional near infrared spectroscopy, and transcranial direct current stimulation (Gorniak et al., 2022). Last but not least, the adiposity proxy measures may provide valuable information beyond the typically used BMI, which has several limitations (Burkhauser & Cawley, 2008;Tomiyama et al., 2016), especially when used in cohorts of older individuals (Bhurosy & Jeewon, 2013;Rothman, 2008). This might be particularly relevant as many open-source brain imaging databases (e.g., ADNI, AIBL, OASIS) include a high percentage of adults who are past midlife.

### 4.3 Limitations and future directions

The bone and adiposity estimations derived from T1-weighted MRI scans are not suitable for clinical use. Moreover, the application of the tool to real-world MR clinical data that lack body composition measures is limited. As demonstrated inFigure S7, downsampled T1-weighted MRI images imitating clinical images can be processed, albeit with lower accuracy. Results obtained from different MR protocols are not directly comparable as structural protocols can vary in (fat-suppression) intensity, which can affect the estimates in various ways. Characterising the nature of these variations and developing robust estimations for MRI protocols with fat suppression is warranted in future studies. Additionally, our proxy BMD measure is highly correlated with head BMD, but only moderately with the BMD of femoral neck, which is a better predictor of fracture risk than head BMD (Kanis et al., 2005). With their relation to high morbidity and mortality in older adults as well as their incidence in AD, fragility fractures present a relevant aspect of ageing (Friedman et al., 2010;Ruggiero et al., 2024). Thus, future studies utilising machine learning might focus on establishing a better predictor of (femoral neck) BMD and fracture risk. Moreover, deep learning might aid in segmenting specific tissue classes, such as muscles, skin, and fat, which could result in an even better (skinfold-like) approximation of an individual’s status related to overall health as well as obesity (Langner et al., 2020;Leong et al., 2024). Altogether, the present study serves as a pilot exploring the potential value of non-brain tissue analyses in the field of neuroimaging. Further studies, possibly extending to other imaging modalities, are necessary to improve and validate the measures.

## Supplementary Material

Supplementary Material

## Data Availability

The data used for this work were obtained from the UK Biobank Resource (Project Number 41655). Due to the nature of the data-sharing agreement, we are not allowed to publish the data. The OASIS-3 dataset is openly available athttps://sites.wustl.edu/oasisbrains/home/oasis-3/. Prior to accessing the data, users are required to agree to the OASIS data use terms (DUT), which follow the creative commons attribution 4.0 licence. The code developed in this study is available on GitHub:https://github.com/robdahn/boney.
