# Supplementary material for: Approximation of bone mineral density and subcutaneous adiposity using T1-weighted images of the human head
Source: Imaging Neurosci (Camb). 2024 Dec 6;2:imag-2-00390. doi: 10.1162/imag_a_00390 (PMC12315763; doi:10.1162/imag_a_00390)
Supplement: Supplementary Material [file imag_a_00390-supp.pdf]

Supplementary material

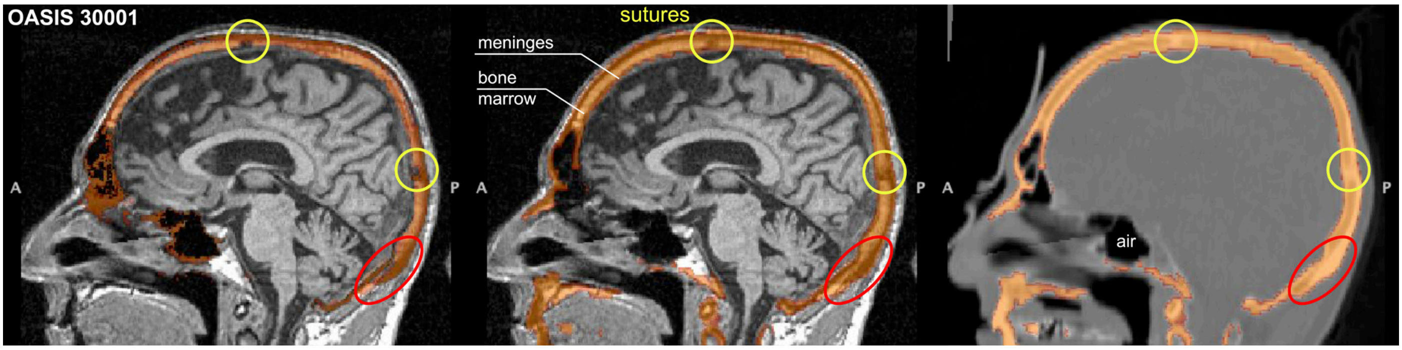

Figure S1. (A) Segmentation result of an OASIS-3 subject for MRI and CT. Overlap of the original SPM MRI segmentation (left) and the CTseg CT segmentation on the MRI (middle) and the co-registered CT images. The general overlap of the MRI and CT image is very good. The oversegmention of the skull on the top of the head could indicate light chemical shift artefacts. Details of the bone structure are visible in both, CT and MRI, where sutures (yellow circles) are darker in MRI but brighter in CT, and the bone marrow is brighter in MRI but darker in CT (also in case of fat suppression) (red).

Table S1. Results of the best predictor estimation in 5 folds.

Fold	Best predictor 1 [variable importance]	Best predictor 2 [variable importance]	Best predictor 3 [variable importance]	Variance explained by the model
1	sROI_bonecortex01 0.053	**main_sBMDH** 0.051	sROI_bonethickness03 0.051	0.62
2	**sROI_bonecortex03** 0.052	vROI_bonethickness01 0.047	sROI_bonecortex01 0.045	0.60
3	**sROI_bonecortex03** 0.052	**main_sBMDH** 0.051	sROI_bonethickness03 0.043	0.60
4	**sROI_bonecortex03** 0.061	**main_sBMDH** 0.056	sROI_bonecortex01 0.055	0.65
5	**main_sBMDH** 0.054	sROI_bonecortex04 0.051	**sROI_bonecortex03** 0.051	0.63

Notes. sROI_bonecortex01: surface-based global bone intensity value, sROI_bonecortex03: surface-based occipital bone intensity value, sROI_bone-cortex04: surface-based right parietal bone intensity value; sROI_bonethickness03: thickness of the occipital bone;
main_sBMDH is the opposite value of the sROI_bonecortex03 (mean occipital bone intensity value);
Variable importance: difference in RMSE of the model using predicted vs. permuted observations (Fife & D'Onofrio, 2023).

Table S2. ICC coefficients from a two-way mixed effect model with absolute agreement for OASIS-3 rescans within 3 months ($p < 0.001$). The images were mostly acquired on different Siemens scanners with slightly different protocols independent of the time point, introducing a slight bias in the measures. The presented results are for the full sample, a sample with identical protocols, and the full sample after harmonisation with ComBat.

Measures	Full sample (N = 157)	Same protocol (N = 63)	Harmonised data (N = 157)
Occipital bone intensity (sROI_bonecortex03)	0.57	**0.95**	0.88
Occipital bone thickness (sROI_bonethickness03)	0.81	**0.83**	0.80
SPM head tissue class intensity (tis_head)	0.50	**0.66**	0.63
Occipital head thickness (sROI_headthickness03)	0.16	**0.53**	0.56
Relative GM volume (rGMV)	0.93	**0.97**	0.92

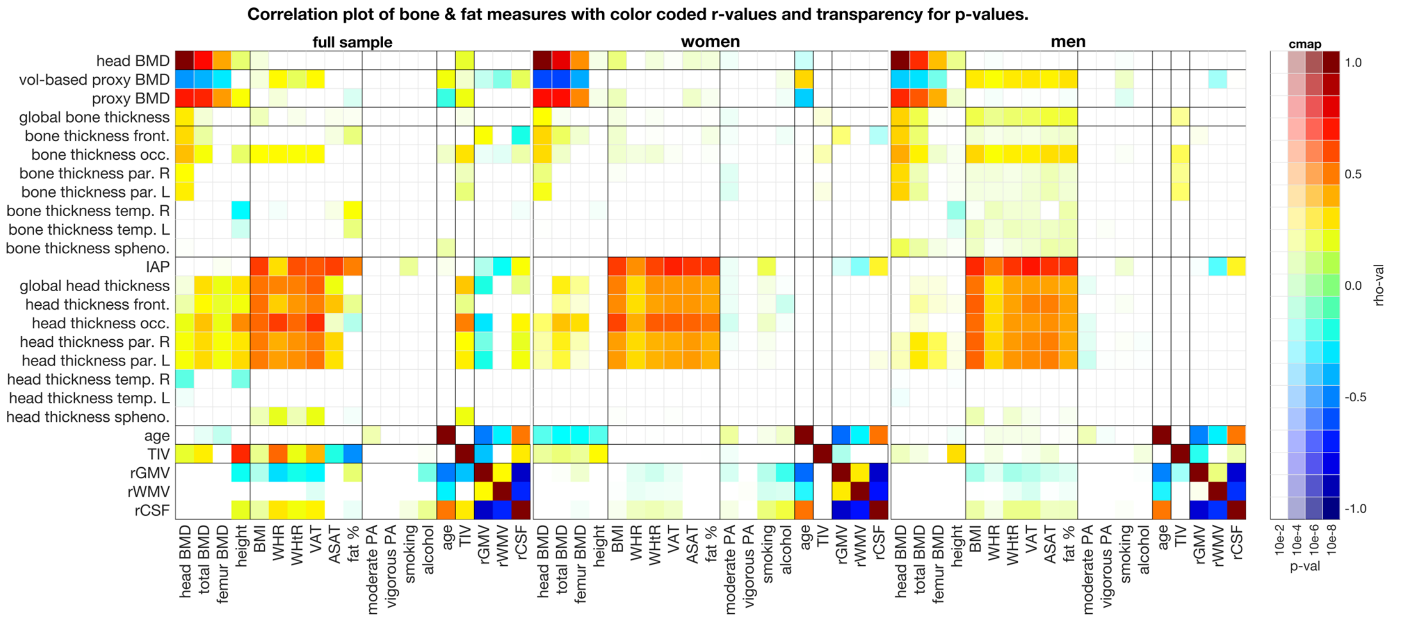

Figure S2. Spearman correlation coefficients with Holm correction for multiple comparisons between the estimated skull BMD proxy, head thickness, and other UKB and brain measures. SPM bone class represents a weighted average of the 3 Gaussian curves estimated by the SPM in the bone class. Vol-based proxy BMD is the volume-derived estimation of the bone intensity in the occipital bone. Proxy BMD is the opposite value of the surface-derived estimation of the bone intensity in the occipital bone.

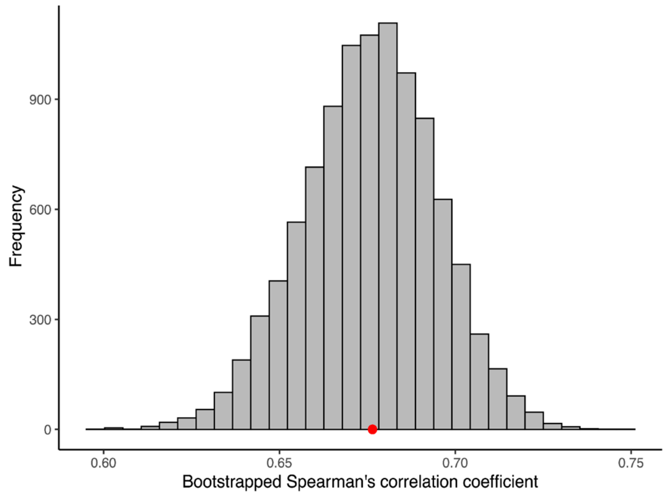

Figure S3. A distribution of bootstrapped Spearman's correlation coefficients between the proxy BMD measure and DXA-derived head BMD. The red dot represents the Bootstrap estimated correlation coefficient (rho = 0.68, *p*<.001, 95% CI 0.64–0.71).

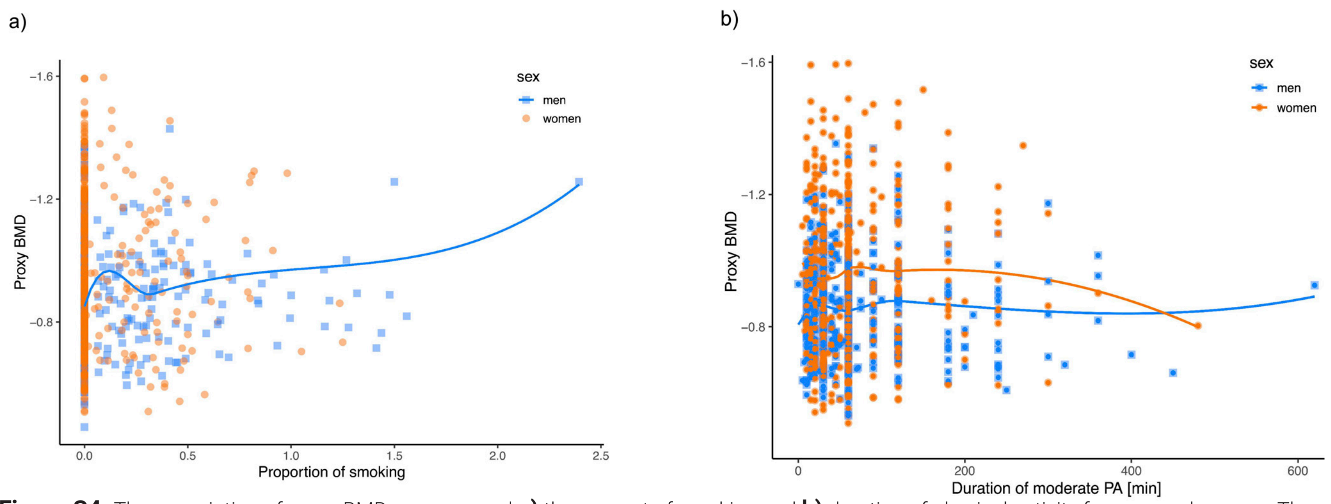

Figure S4. The association of proxy BMD measure and **a)** the amount of smoking and **b)** duration of physical activity for men and women. The negative effects of smoking are more evident in men, whereas positive effects of moderate physical activity are more prominent in women.

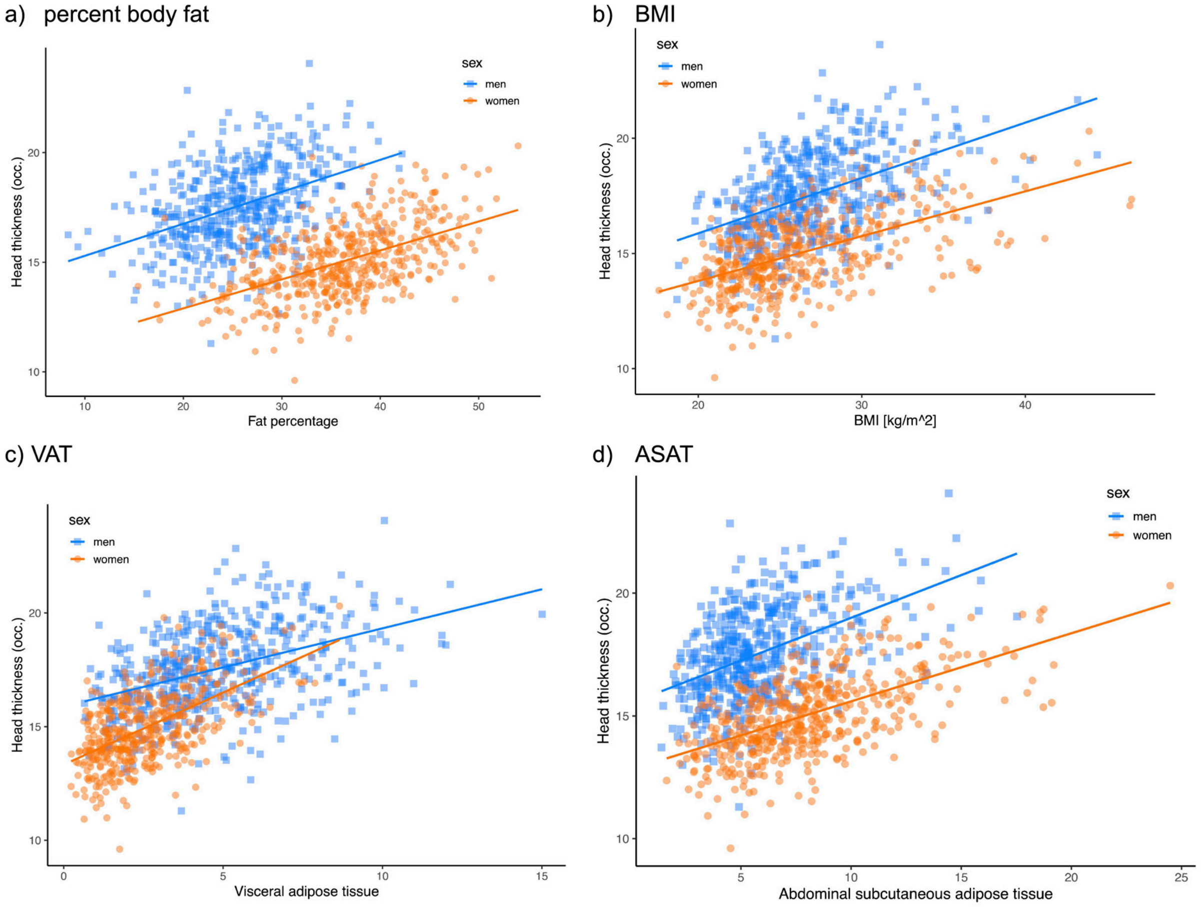

Figure S5. The association between head thickness estimation in the occipital part and **a)** percent body fat, **b)** BMI, **c)** VAT (visceral adipose tissue), and **d)** ASAT (abdominal subcutaneous adipose tissue).

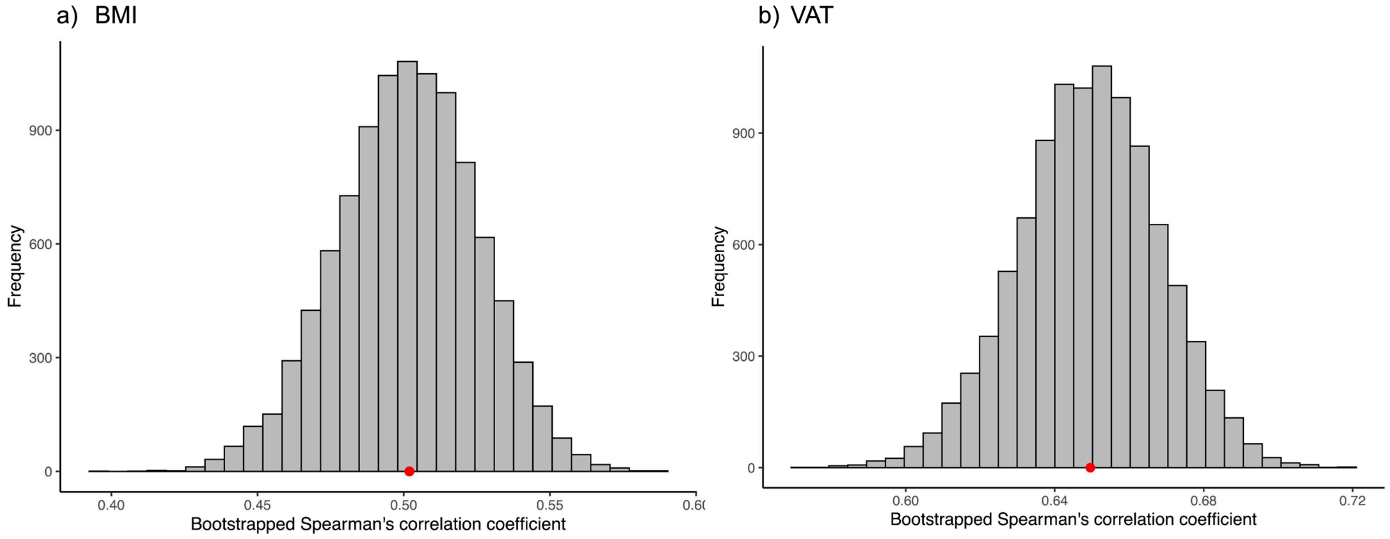

Figure S6. Bootstrapped Spearman's correlation coefficients between the head thickness approximation and the **a)** BMI (rho = 0.50, p<.001, 95% CI: 0.45–0.55) and **b)** VAT (rho = 0.65, p<.001, 95% CI: 0.61–0.69).

Downsampled UKB MRI images (resolution: 5x1x1 mm)

To test the potential application to clinical data, we downsampled the original T1-weighted images from the UKB to imitate clinical data with a sagittal orientation and a slice thickness of 5 mm. A two-way-mixed effects model testing for consistency showed a good agreement between the values extracted in both cases (ICC = .93, p<.001, 95% CI 0.92–0.94).

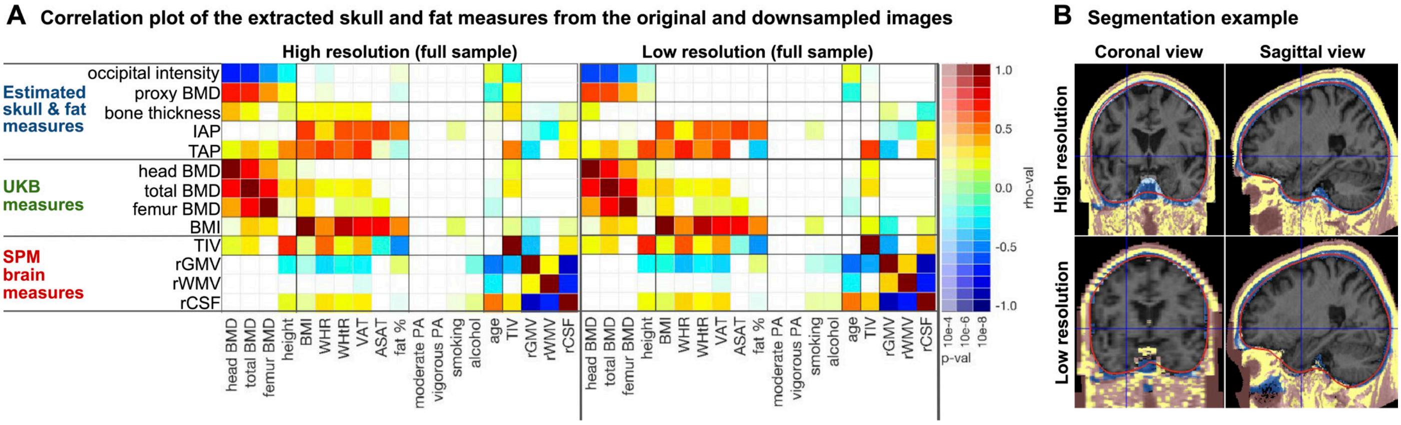

Figure S7. A) Comparison of Spearman correlation coefficients with Holm correction for multiple comparisons between the estimated skull BMD proxy, bone thickness, intensity-based adiposity proxy, thickness-based adiposity proxy and other UKB and brain measures obtained from the original images (left) and the downsampled images (right). The Spearman correlation coefficient between our BMD proxy measure and the DXA-derived head BMD on the downsampled images was 0.62, p<.001. The correlation of the intensity-based- and thickness-based adiposity proxy with BMI was 0.60 and 0.44, respectively. **B)** Comparison of segmentation for the 1 mm (above) and 5 mm slice thickness (below) for a single subject from the UKB (Reproduced by kind permission of UK Biobank ©).
